# Standardizing Workflow, Patient Care, and Reimbursement for Radiology Second Opinions

**DOI:** 10.1007/s10278-025-01572-w

**Published:** 2025-06-24

**Authors:** Akhil Dhamija, Matthew Crain, Mohammed Ismail, Clayton R. Taylor, Luciano M. Prevedello, Hillary M. Kummer, Daria Blanton, Robin Weppler, Pari V. Pandharipande, Zarine K. Shah

**Affiliations:** Department of Radiology, The Ohio State University Medical Center, Columbus, USA

**Keywords:** Billing, Second Opinion Interpretation, Standardization, Relative Value Unit, Referral and Consultation, Diagnostic Imaging

## Abstract

Second opinions in radiology are crucial for accurate diagnosis and patient management but often lack standardization, leading to inefficiencies and suboptimal reimbursement. We redesigned the second opinion workflow leveraging existing tools in the EMR. Summary statistics as well as post-implementation surveys were conducted among ordering providers and radiologists to gauge the process. Reimbursement data from August 2021 to October 2022 was also analyzed. The Abdomen division accounted for 47% of second opinion exams, followed by Neuroradiology at 26%, Chest at 17%, Nuclear Medicine at 9%, and MSK at 1%. CT scans comprised 64%, MR 26%, NM 9%, and US 1%. Survey participants reported an improvement in user-friendliness ratings for the second opinion process from 2 to 4.4 (*p* < 0.07), with substantial positive trends among ordering providers (Cohen’s *d* = 2.00). Satisfaction with perceived timeliness of report availability was high across both groups, and radiologists perceived workflow efficiency improved from 2.7 to 4 (*p* = 0.036). The new workflow achieved a 91.9% collection success rate. Standardizing the second opinion workflow enhances operational efficiency, user satisfaction and reimbursement, thereby improving patient care.

**Supplementary Information:**

The online version contains supplementary material available at 10.1007/s10278-025-01572-w.

## Background

Second opinions in radiology are essential for providing subspecialized expert interpretation, addressing discrepancies in findings, answering specific clinical questions, and comparing prior examinations. Despite their importance, the process of obtaining second opinions has traditionally been inconsistent, informal, and often ill-defined [1, 2].

Second opinion reporting in radiology can take many forms at different institutions and best practices are not standardized. These non-standardized informal processes have several pitfalls including insufficient or absent documentation within the Electronic Medical Record (EMR), miscommunication of findings, adversely affecting patient care with potential downstream legal consequences, inefficient workflows, resulting in increased workload for both clinical services and radiology departments, the risk of repeat imaging, leading to higher radiation exposure for patients and increased financial costs and the absence of a systematic approach to account for radiologists’ time and effort.

Prior to this streamlined second opinion process, requests for our departments Radiologist’s interpretation of studies done at outside institutions were informal and either done verbally over the phone, in the reading room as a consult or at case conferences. This caused interruptions to workflows and disruptions in the reading room. These consultations were then manually documented by the clinical team, which often led to limited or incorrect interpretation of the Radiologist’s findings in the EMR. In other situations, radiologists provided focused, unstructured reads as addenda to the medical record, a process that was poorly defined and inconsistently applied. This process of documenting an addendum was cumbersome, only addressed a single finding, and was difficult to track. The addendum, when provided, did not show up on the patient’s list of study reports and had to be manually searched. The steps involved in issuing the addendum involved multiple emails between the reading room assistants and a team of Radiologists with a lack of accountability often resulting in delayed communication of findings. The Radiologist's input and time spent on these activities was unaccounted for as there was no mechanism for coding or billing.

Ideally, to establish a sustainable second opinion process, it is crucial to prioritize efficiency and ensure Radiologists’ efforts are properly captured and reimbursed. While second opinions are widely utilized, detailed billing practices remain poorly documented in the literature [4, 5]. Despite the high demand for these services, particularly at large academic medical centers, there is no standardized or formalized approach that also considers billing and reimbursement of professional expenses. Although a generic Current Procedural Terminology (CPT) code exists for radiology second opinions, it fails to account for imaging modality or study specifics, potentially leading to discrepancies. To address this, we aligned our second opinion process for outside imaging studies with our internal workflow. This included using the same dictation templates and ensuring the specific CPT code for the study performed was accurately captured, coded, and billed.

This manuscript details our institution’s efforts to optimize the second opinion process through process redesign, in line with the American College of Radiology’s (ACR) recommendation for policies on reporting outside studies and documenting all consultations [6].

## Methods

This project was submitted to the Institutional Review Board (IRB) and was deemed exempt due to its minimal risk.

### Design of the New Workflow

**Fig. 1:**
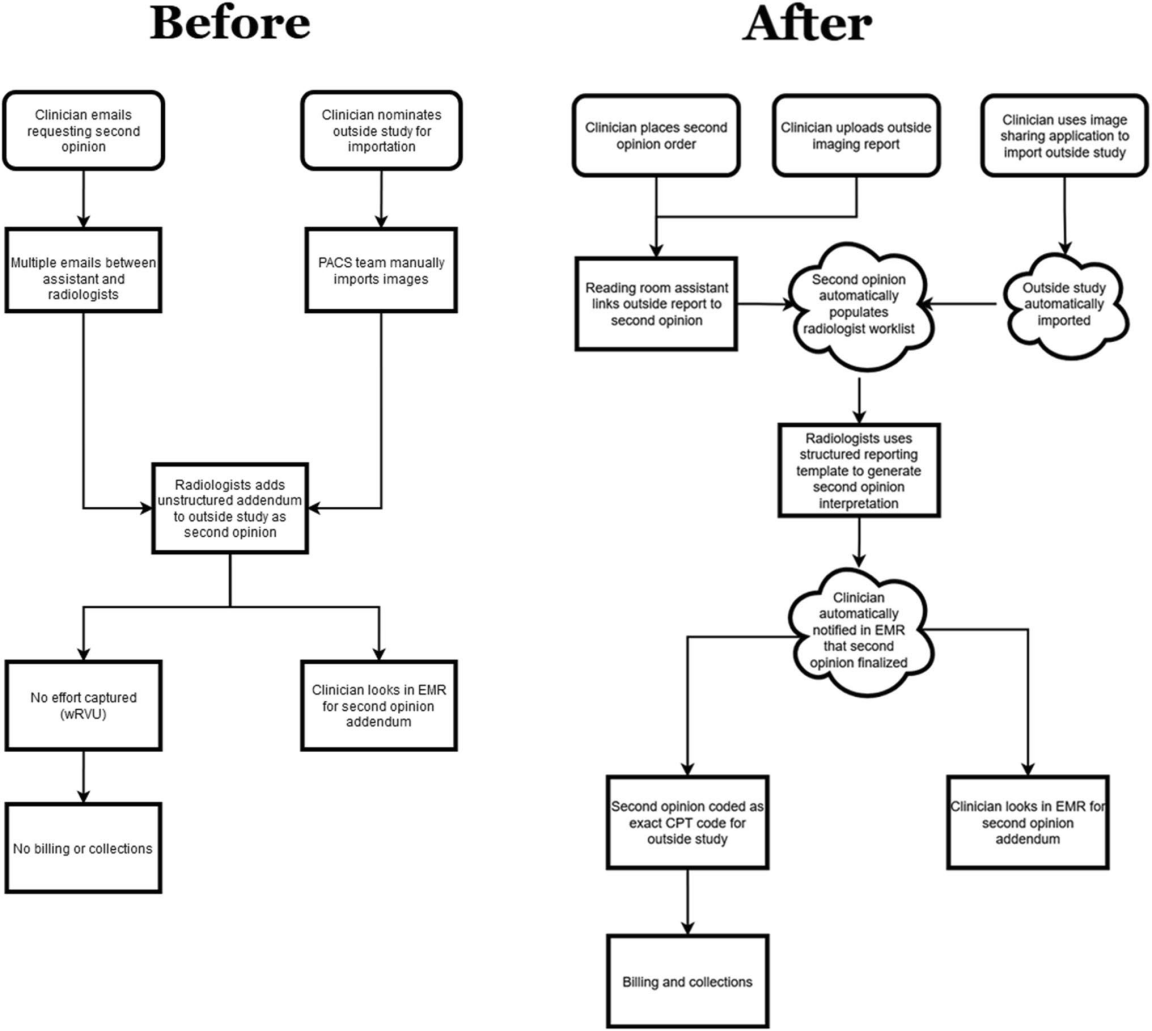
Detailed workflow schematic before and after implementation

To address the inefficiencies of the system that issued addenda, several opportunities for improvement were identified (Fig. 1). The redesigned process for ordering a second opinion starts with automating the importation of outside imaging studies using a patient reconciliation tool (AMBRA, Intelerad, New York, NY) that harmonizes patient identifiers and links studies to the appropriate patient’s chart. A second opinion order is then placed, and administrative staff (reading room assistants) monitor a worklist for second opinions, ensuring that the request meets departmental criteria. These criteria include (a) outside report available for Radiologists’ review, (b) indication that clearly states medical necessity for second opinion and (c) outside imaging study performed within a specific time-frame—30 days initially (pilot phase of the project)—and then extended to 3 months after the initial phase. Once these steps are completed, the study automatically populates a specialty-specific Radiologist worklist, and the reporting process is conducted similarly to in-house studies, using the same reporting software and reporting templates to document findings as those for studies done at our institution. Using standardized reporting templates and having an indication of medical necessity for the second opinion together allows for the original Work Relative Value Unit (wRVU) of the CPT code of the outside study to be documented and used for billing.

These workflow changes were implemented in August 2021 for all divisions within our department except Breast Imaging (The division of Breast Imaging has a separate workflow for second opinions which is optimized to the specific needs of breast surgeons and patients). The initial limited roll-out was a success and allowed for fine tuning of the process and workflow modifications, ultimately leading to the extension of the eligibility timeline for outsides studies from 1 to 3 months in April 2022. The requirement of outside reports for the imaging studies was put in place so that our Radiologists had all the available information, including knowledge of any priors that were used for comparison and any additional clinical or imaging data that would allow for a more holistic report. This is especially valuable for patients who are transferring into our tertiary care facility from other locations and community sites.

### Data Collection and Analysis

As a departmental initiative to understand workflows following the transition to the new workflow at the end of July 2021, a database was maintained. For this project’s purposes, August 2021–October 2022 was used for analysis. This database, created for departmental quality improvement and request tracking, was implemented to include ordering and interpreting physician information. After the new workflow was implemented, this database was expanded to capture reimbursement data (denied or approved), reasons for reimbursement denial, and rationale for non-fulfillment of second opinion reads. A range of summary statistics was calculated, along with detailed metrics to include sub-specialty specific breakdowns, exam type categorization, distribution of collections, collection success rate (defined by successful coding, billing, and collection processes), and the overall collection rate (representing the proportion of amounts collected relative to the total billed, adjusted for institutional contractual agreements).

Additionally, two 5-min surveys (Appendix 1 & 2) were conducted in April 2024 for a 4-week period to assess the utility of the new second opinion process: one for ordering providers to evaluate the functionality of the workflow and its impact on patient care, and second for Radiologists to gather feedback on their experience with the new workflow. The survey was conducted using Research Electronic Data Capture (REDCap) after obtaining informed consent from participants and the data above was analyzed using simple summary statistics in excel.

This data collection and analysis approach provides insights into the efficiency, clinical impact, and reimbursement structure of the redesigned workflow.

## Results

Six hundred seventeen second opinion reports were analyzed, with study type and sub-specialty distribution and billing data summarized in Tables 1 and 2. Of the 617 total exams, 553 (89.62%) were successfully reimbursed, while 54 (8.75%) instances of failure to reimburse and 10 (1.62%) instances of no charges were recorded. This resulted in a reimbursement success rate of 91.9% and an overall collection rate (amount collected with respect to total billed, considering institutional contractual agreements) of 36%. The exams we were unable to collect were primarily due to authorization issues, with 31 out of 54 (57.41%) cases lacking the necessary authorization. Additionally, 18 (33.33%) exams were not able to be collected due to patients'deductible/patient obligations or self-pay situations. Two (3.70%) exams were not able to be collected due to patients’ uninsured status and for 3 (5.56%) exams reasons for inability to collect were unclear.

**Table 1:** Breakdown of number of exams and collection rate by section. The total does not include exams where no charges were made

Sub-specialty division	Modality	Number of exams	% of total exams	Average collection rate
Abdomen		287	47.28%	0.3773
	CT	199	32.78%	0.3757
	MR	81	13.34%	0.3916
	US	7	1.15%	0.258
Chest		103	16.97%	0.3532
	CT	103	16.97%	0.3532
MSK		7	1.15%	0.2721
	MR	7	1.15%	0.2721
Neuro		158	26.03%	0.364
	CT	91	14.99%	0.3274
	MR	67	11.04%	0.4136
Nuclear medicine		52	8.57%	0.3726
	PET	41	78.85%	0.3394
	Bone scan	11	21.15%	0.4713
Total		607	100.00%	0.3681

**Table 2:** Biling summary metrics

Overall metrics	
Total submitted for reimbursement	553
Total failure to reimburse	54
Total no charges	10
Total exams (including no charges)	617
Reimbursement denial rate	8.90%
Average overall collection*	36%

^*^Collection rate – amount collected with respect to total billed, considering institutional contractual agreements

The Abdomen division accounted for the largest portion of exams, comprising 47.28% of the total, with an average collection rate of 37.73%. The Neuroradiology division represented 26.03% of exams, with an average collection rate of 36.40%. The Chest division contributed 16.97% of the exams, with an average collection rate of 35.32%. The Nuclear Medicine division accounted for 8.57% of the exams, achieving an average collection rate of 37.26%. The Musculoskeletal (MSK) imaging division had the smallest share at 1.15% of the exams, with an average collection rate of 27.21%.

Across all sections, Computed Tomography (CT) exams constituted the majority at 64.29% of the total, with an average collection rate of 35.97%. Magnetic Resonance (MR) exams made up 26.46%, with an average collection rate of 37.56%. Ultrasound (US) exams accounted for 1.15%, with a collection rate of 25.80%. Nuclear Medicine (NM) exams represented 8.57% of the total, with a collection rate of 37.26%.

The primary reasons for the inability to provide a second opinion report were incomplete image data and the lack of clinical context/question, each cited 4 times. Other reasons included inappropriate scan quality (2 times), and individual instances of reports not being indicated, dealing with very old studies (where a more recent study was available for clinical evaluation), and lack of availability of outside reports (Table 3).

**Table 3:** Reason(s) for inability to provide a second opinion report

Reason(s) for inability to provide second opinion report	
Incomplete image data	4
Clinical context/question not available	4
Quality of scans not appropriate	2
Study not indicated	1
Very old study (with more recent prior)	1
Outside report not available	1

### Survey Data

A total of 67 surveys were sent out, 31 to ordering providers and 36 to Radiologists. A total of 27 (40%) surveys were returned, with a survey response rate of 29% (9/31) from ordering providers, 33% (12/36) from Radiologists, and 9% (6/67) were incomplete responses. The user-friendliness of the process before and after the implementation was evaluated among both groups. Ordering providers reported improvement with mean user-friendliness ratings increasing from 2.0 before the intervention to 4.4 after the intervention (*p* = 0.074), however did not reach statistical significance due to low sample size (3). Radiologists’ efficiency ratings between the old and new second opinion workflows revealed a statistically significant improvement in perceived efficiency which increased from 2.71 to 4 (*p* = 0.036) and user-friendliness post-implementation (*p* = 0.017) with a higher sample size (7). Radiologists unanimously agreed that the redesigned process made it easier to provide a full report for cases eligible for a second opinion. The effect size for the improvement in user-friendliness among ordering providers was substantial, with Cohen’s *d* calculated at 2.00 for ordering providers and 1.23 for Radiologists, indicating a large effect size.

The new workflow’s perceived impact on patient care was also assessed. Ordering providers expressed strong support for the workflow’s ability to reduce unnecessary imaging, with most giving the highest possible score. This resulted in a median rating of 5.0, with only 1/9 respondents rating it below 4. Radiologists provided similarly positive ratings, reflecting the belief that the workflow would improve patient care and help avoid unnecessary imaging. Satisfaction with the timeliness of second opinion reporting was similarly evaluated. Ordering providers reported a median satisfaction score of 5.0 on a scale of 1 to 5 with only 2/9 responses below a score of 4, reflecting high satisfaction with the new workflow. Radiologists echoed this sentiment, with similarly high satisfaction scores.

## Discussion

The redesigned workflow for obtaining second opinions in Radiology at our institution has led to operational improvements and a positive impact on patient care. The process redesign focused on standardizing and formalizing the previously informal and inconsistent second opinion workflow. This structured approach addressed key issues such as inadequate documentation, miscommunication, and inefficiencies that plagued the earlier workflow. In our experience, the success of these improvements is related to seamless integration into our routine reporting processes. Outside studies are easily identifiable but are viewed on the same picture archiving and communication system (PACS) system, ensuring consistency. Utilization of standardized templates for reporting, tailored to the study type, allowed relatively easy adoption of this new workflow in a busy clinical practice with minimal disruption. Reports are available under the second opinion order and are integrated into the EMR, ensuring continuity of care and visibility for the entire care team.

Another very relevant outcome of this redesign, important for sustainability of the process, was the ability to consistently bill for these studies with consistent reimbursement across various study types. The reimbursement success rate was 91.9%, equivalent to the rates observed for internal imaging studies performed at our institution. This was a promising result and allowed continued use of this process and maintained financial viability. One of the reasons for variation in collection rates across sections, particularly the lower rate in the MSK section, can be attributed to a smaller sample size in that category. The successful integration of modalities like CT and MR into the workflow demonstrates the system’s capability to handle high-volume processes efficiently.

For continued success and additional process improvement, we realize that future efforts can be focused on improving authorization processes. Specifically, implementing more rigorous pre-verification protocols to enhance communication with referring providers, ensuring all necessary authorizations are secured before second opinion exams.

The information gathered from the surveys supports the success of the workflow redesign. Both ordering providers and Radiologists reported improvements in user-friendliness, with ordering providers showing a significant increase in their ratings and a statistically significant increase in ratings from the Radiologists. Satisfaction with the perceived timeliness for obtaining second opinions was also notably high among both groups. Radiologists also reported a perception of improved efficiency as compared to the more cumbersome old process.

The positive impact on patient care was evident, as both ordering providers and radiologists recognized the benefits of the new workflow in reducing unnecessary imaging and enhancing overall care quality. However, it is important to note that second opinions can incur real costs for patients, potentially leading to financial burdens [7]. That said, these costs may be partially offset by charging only for the wRVU for professional component rather than the technical and professional cost of a potential repeat study. We acknowledge that there are limitations in our study. The small sample size and incomplete survey responses may limit the generalizability of the findings. Additionally, the inability to perform direct paired analysis for Radiologists restricts a deeper comparative analysis. The short study period selected may not fully capture long-term trends and outcomes associated with the workflow redesign. This process, however, is successfully utilized currently at our institution and this workflow redesign has been broadly successful, with benefits in operational efficiency and patient care.

## Conclusion

The workflow redesign has demonstrated promising results in improving operational efficiency, user satisfaction, and patient care. The promising collection rates, enhanced user-friendliness, and high satisfaction with perceived timeliness of reporting underscore the success of the new process. The improvements observed in both operational metrics and user satisfaction highlight the value of a standardized and formalized approach to managing second opinions in radiology. Continued monitoring and refinement of the workflow will be essential to maintaining and further enhancing these positive outcomes.

## Supplementary Information

Below is the link to the electronic supplementary material.

Supplementary file1 (PDF 115 KB)

## Data Availability

Data available upon request due to restrictions in privacy issues. The data presented in this study are available upon request from the corresponding author.
