# Supplementary material for: Standardizing Workflow, Patient Care, and Reimbursement for Radiology Second Opinions
Source: J Imaging Inform Med. 2025 Jun 24;39(2):1094–9. doi: 10.1007/s10278-025-01572-w (PMC13103147; doi:10.1007/s10278-025-01572-w)
Supplement: Supplementary file 1 — Supplementary file1 (PDF 115 KB) [file 10278_2025_1572_MOESM1_ESM.pdf]

Appendix 1: Radiologist Survey

Radiologist Survey

What division of radiology do you belong to?

____

Did you provide second opinion reports or addendums to imaging studies prior to the implementation of the formalized second opinion workflow (July 2021)?

◯ Yes
◯ No

On a scale of 1-5 how would you rate the efficiency of the old workflow for second opinions?

1 (very poor) 3 (neutral) 5 (very good)

(Place a mark on the scale above)

In your opinion, has the redesigned process made it easier to provide a full report for the cases that are eligible for a second opinion?

◯ Yes ◯ No

On a scale of 1-5 how would you rate the efficiency of the new workflow for second opinions?

1 (very poor) 3 (neutral) 5 (very good)

(Place a mark on the scale above)

Have you come across situations where you were unable to provide a second opinion report in this new process?

◯ Yes ◯ No

What is the reason(s) you were unable to provide a second opinion report? (select all that apply)?

☐ Images not complete/available
☐ Outside report not available
☐ Quality of scans not appropriate
☐ Clinical context/question not available
☐ Other (describe)

Please describe

____

On a scale from 1-5 please indicate how the reading room support staff have enabled the workflow in this new second opinion process?

1 (very poor) 3 (neutral) 5 (very good)

(Place a mark on the scale above)

Have you found situations where there is a clinically significant discrepancy between your interpretation and the outside radiologist interpretation?

◯ Yes ◯ No

Have you found clinical utility of having an OSU radiology report for the scans that are done at non-OSU facilities when reading a follow-up study done here? (e.g. you found a report on a relevant prior done elsewhere and were able to read that report while reading the current scan for the patient)

◯ Yes ◯ No

On a scale of 1-5 how beneficial is it to have a report attached to a relevant prior study compared to a note about a phone conversation or curbside consult?

1 (not beneficial)	3 (neutral)	5 (very beneficial)

(Place a mark on the scale above)

Appendix 2: Ordering Provider Survey

Ordering Provider Survey

What service do you belong to?	__________________________
Have you requested a second opinion on an imaging study prior to the implementation of the formalized second opinion workflow (prior to July 2021)?	◯ Yes<br>◯ No
On a scale of 1-5 please rate how user friendly the old process was while placing the order in EPIC for the second opinion?	1 (very poor) 3 (neutral) 5 (very good)<br>*(Place a mark on the scale above)*
On a scale of 1-5 please rate how user friendly the new process is while placing the order in EPIC for the second opinion?	1 (very poor) 3 (neutral) 5 (very good)<br>*(Place a mark on the scale above)*
On a scale of 1-5 how satisfied are you satisfied with the turnaround time to get a formal second opinion report?	1 (very unsatisfied) 3 (neutral) 5 (very satisfied)<br>*(Place a mark on the scale above)*
Based on your practice patterns what is the ideal time window to allow for second opinion reads? (e.g. up to 2 months or up to 5 months; enter maximum acceptable)	__________________________<br>(months max)
On a scale of 1-5 how helpful was this process in improving patient care?	1 (not helpful) 3 (neutral) 5 (very helpful)<br>*(Place a mark on the scale above)*
On a scale of 1-5 how helpful was this process to avoid additional unnecessary imaging for your patients?	1 (not helpful) 3 (neutral) 5 (very helpful)<br>*(Place a mark on the scale above)*
